# 

**Published:** 1920-03

**Authors:** 


### ANNOUNCEMENTS

The Michigan State Dental Society will hold its next regular meeting at the Hotel Statler, Detroit, Mich., April 12, 13 and 14, 1920.

The chairman of the local arrangements is Wm. H. Waller, 613 Washington Arcade, Detroit.—Claude S. Larned, Secy., 614 Post Bldg., Battle Creek, Mich.

---

The next regular meeting of the Kentucky State Dental Association will be held in Louisville, April 7, 8 and 9, 1920.

A program of unusual interest has been planned. Address all correspondence to W. M. Randall, Secy., 1935 Second St., Louisville, Ky.

---

The fifty-sixth annual meeting of the Illinois State Dental Society will be held in Chicago, beginning Monday, March 22, 1920, and continuing four days. The headquarters will be at the Congress Hotel, where the sessions, clinics and exhibits will be housed.—J. P. Lüthringer, Secy., Peoria, Ill.

---

The next meeting of the West Virginia State Dental Society will be held in Charleston on April 14, 15 and 16, 1920.

